# Characterization of Chemically-Induced Endogenous Retroviral Particles in the CHO-K1 Cell Line

**DOI:** 10.3390/v17111408

**Published:** 2025-10-23

**Authors:** Nicholas B. Mattson, Trent J. Bosma, Yamei Gao, Sandra M. Fuentes, Pei-Ju Chin, Arifa S. Khan

**Affiliations:** Laboratory of Retroviruses, Division of Viral Products, Office of Vaccines Research and Review, Center for Biologics Evaluation and Review, U.S. Food and Drug Administration, Silver Spring, MD 20993, USA; nmattson91@gmail.com (N.B.M.); tjbosma@gmail.com (T.J.B.); yamei.gao@fda.hhs.gov (Y.G.); sandra.fuentes@fda.hhs.gov (S.M.F.); pei-ju.chin@fda.hhs.gov (P.-J.C.)

**Keywords:** Chinese hamster ovary cells (CHO cells), retrovirus-like particles (RVLPs), endogenous retroviruses, 5-iodo-2′-deoxyuridine (IdU), 5-azacytidine (AzaC), virus induction assays, product-enhanced reverse transcriptase assay (PERT), transmission electron microscopy (TEM), high-throughput sequencing (HTS)

## Abstract

The Chinese hamster ovary K1 cell line (CHO-K1) constitutively produces retroviral-like particles (RVLPs) containing reverse transcriptase (RT) activity, which, thus far, have not been shown to be infectious. Since infectious retroviruses have been reported in other rodent species, this study was undertaken to investigate the presence of latent, infectious, endogenous retroviruses (ERVs) in CHO-K1 cells by using chemical induction assays and detection of activated virus using the highly sensitive, product-enhanced RT (PERT) assay, with subsequent infectivity analysis in cell lines of different species, including human. The results demonstrated activation of A-type and C-type retroviral particles based on transmission electron microscopy and increased production of cell-free RT-particles after treatment of the cells with 5-iodo-2′-deoxyuridine and 5-azacytidine, which was greater with dual treatment than with each inducer alone. Induction of A- and C-type particles was confirmed in dual-drug-treated CHO-K1 cells by long-read high-throughput sequence (HTS) analysis. Infectivity studies performed by inoculating human A549, HEK-293, and MRC-5 cells; African green monkey Vero cells; *Mus dunni* cells; and CHO-K1 cells with supernatant containing RT-particles from dual-treated CHO-K1 cells indicated the absence of a replicating retrovirus in supernatant from extended cell culture using the PERT assay. Furthermore, short-read HTS analysis did not show evidence of integration of retroviral sequences in inoculated A549 and 293 cells. The overall results showed no evidence for latent, infectious, endogenous RVLPs in CHO-K1 cells.

## 1. Introduction

The Chinese hamster ovary K1 cell line (CHO-K1) is derived from an ovarian biopsy of an adult Chinese hamster (*Cricetulus griseus*) [1] and is widely used for large-scale production of proteins in the biopharmaceutical industry, such as recombinant products [therapeutics, vaccines, virus-like particles (VLPs), and gene therapy products] [2,3,4,5]. The use of the CHO-K1 cell line is popular since the cells are refractory to infection by most human viruses and are able to post-translationally modify proteins in a manner similar to human cell lines [3,4]. However, like many rodent cell lines, CHO-K1 cells express endogenous retrovirus-like particles (RVLPs), which have been characterized morphologically as intracytoplasmic A-type particles [6,7,8,9,10,11,12] related to the *Alpharetrovirus genus* and budding C-type particles [12,13,14,15,16,17,18,19,20], corresponding to the *Gammaretrovirus genus*. Two other endogenous retrovirus particles that have been reported in hamster cells are B-type particles, which have been described as mature intracytoplasmic A-type particles in CHO cells [10] or enveloped mature A-type particles [21], and R-particles that have been seen with 5-azacytidine treatment of Syrian hamster cells [22]. The A-type particles in Syrian hamsters are closely related to B-type mouse mammary tumour virus (MMTV) and D-type squirrel monkey retrovirus (SMRV) [23]; however, the relatedness between the A-type particles in Chinese hamsters and those in Syrian hamsters is unclear [24].

The RVLPs produced from CHO cells have been reported to be non-infectious [12,18,19]. It should be noted that although A-type particles are expected to be replication-defective since they are the result of an ancient insertion that has degraded over time due to small insertions and deletions, with little to no evidence of an intact *gag*, *pol*, or *env* ORF [11,24,25], they can be functionally active, resulting in disruption of normal cellular processes by insertional mutagenesis [25,26,27,28,29,30,31]. Characterization of C-type retroviral sequences in the CHO-K1 cell genome has indicated that the majority are replication-defective due to deletions or point mutations; however, there is at least one locus that has the potential for encoding a virus [15,17,32]. This provirus has an overall 64% homology to the Moloney murine retrovirus [15,16,17] and an envelope gene that is closely related to amphotropic murine leukemia viruses (MLVs), which are known to have a wide host range, including human cells [16,33,34,35,36,37,38]. The majority of the early studies were focused on characterization of the retroviral sequences or particles in CHO cells, and some infectivity studies reported an absence of an infectious virus [12,18,19]. To investigate the presence of a latent, infectious endogenous retrovirus in the CHO-K1 cell line, we applied chemical induction strategies for activating RVLPs to simulate the stressful culture conditions that could potentially activate latent viruses during large-scale biomanufacturing. Additionally, we used long-read, high-throughput sequencing (HTS) to characterize the induced viruses and performed extended infectivity assays using a variety of target cell lines combined with the highly sensitive PCR-enhanced RT (PERT) assay to evaluate for the presence of a replicating retrovirus and short-read HTS to investigate for potential virus integration. The overall strategy can be applied for the general evaluation of endogenous infectious retroviruses in cell lines used for research and in manufacturing.

## 2. Materials and Methods

### 2.1. Cell Lines

Cell lines were obtained from the American Type Culture Collection (ATCC; Manassas, VA, USA). A master cell bank was prepared at passage 5 for the CHO-K1 studies (Chinese hamster ovary cell line, catalogue no. CCL-61, lot 70029706, passage unknown). The target cells for the infectivity studies were 293 [HEK-293] (human embryo kidney cell line, catalogue number CRL-1573, lot 3989286, passage 36); A549 (human lung epithelial cell line; catalogue no. CCL-185, lot no. 3285902, passage 76); Vero (African green monkey kidney cell line; catalogue no. CCL-81, lot no. 3645301, passage 120); *Mus dunni* (wild mouse fibroblast cell line, CRL-2017); and MRC-5 (human fetal lung fibroblast diploid cell line, catalogue no. CCL-171, lot no. 3314603; passage 16).

All cell lines were grown in complete media supplemented with 10% fetal bovine serum (FBS) (heat inactivated at 56 °C for 30 min; HyClone, Logan, UT, USA, catalogue no. SH30071.03), and penicillin and streptomycin, 100 units/mL and 100 μg/mL, respectively (Quality Biological, Inc., Gaithersburg, MD, USA, catalogue no. 120-095-721). CHO-K1 cells were grown in F12K complete medium (Gibco; Grand Island, NY, USA, catalogue no. 21127-022); A549 and *Mus dunni* were grown in Dulbecco’s Modified Eagle Medium (DMEM) complete medium (Gibco, catalogue no. 11995-065) with added 2 mM L-glutamine (Quality Biological, Inc., catalogue no. 118-084-721); and 293, Vero, and MRC-5 were grown in Eagle’s Minimum Essential Medium (EMEM) modified complete medium (Gibco, catalogue no. 11095-080) with added 1× non-essential amino acids (Quality Biological, Inc., catalogue no. 116-078-721), 2 mM L-glutamine, and 1 mM sodium pyruvate (Quality Biological, Inc., catalogue no. 116-079-721).

### 2.2. Growth Curve and Population Doubling Time

To determine the optimum number of cells for obtaining a sigmoidal growth curve, CHO-K1 cells (2.5 × 10^5^, 3.33 × 10^5^, 5.0 × 10^5^, 6.5 × 10^5^, and 7.5 × 10^5^) were seeded in 25 cm^2^ flasks in a total volume of 5 mL of F12K complete medium and observed for 72 h for reaching 90–95% confluence. Cells were counted using a Nexcelom Cellometer Spectrum counter, with live cells and dead cells being differentiated by staining using ViaStain AO-PI solution (Nexcelom; Laurence, MA, USA; catalogue no. CS2-0106-25 mL). Population doubling time (PDT) was calculated as 1/*k* = *T* (where *T* = PDT) from the linear curve in the log phase using the formula *N* = *N*_0_2*^kt^* (*N* = total viable cell number at end time *t*; *N*_0_ = total viable cell number at initial time *t*_0_; *t* = hours from *N*_0_ to *N*; and *k* = regression constant) [39,40].

### 2.3. Drug Dose Evaluation and Virus Induction Assays

Chemicals used for inducing endogenous retroviruses were 5-iodo-2′-deoxyuridine (IdU; Sigma Aldrich, St. Louis, MO, USA, catalogue no. 17125-25G; stock solution 50 mg/mL dissolved in 1 N NH_4_OH) and 5-azacytidine (AzaC; Sigma Aldrich, catalogue no. A1287; stock solution 1 mg/mL, dissolved in water).

To initially determine the optimum virus induction conditions, CHO-K1 cells were treated for 36 h (1.7 PDT) by adding different concentrations of AzaC (2.5, 5.0, 7.5, and 10 µg/mL) or IdU (10, 30, and 50 μg/mL) at 16 h and 24 h post-plating the cells (5 × 10^5^ cells in 25 cm^2^ flasks in a total volume of 5 mL F12K complete medium). Induction of endogenous retroviruses was evaluated by analyzing cell-free supernatant using the PERT assay (described below), and drug cell toxicity was visualized by light microscopy.

Based on the results for optimizing virus induction, cells were seeded for 16 h before drug treatment with 2.5 μg/mL AzaC, 30 μg/mL IdU, and combined AzaC and IdU (2.5 μg/mL and 30 μg/mL, respectively). Untreated cells were included as a control. For each condition, four 25 cm^2^ flasks were seeded with 5 × 10^5^ total cells. After 36 h, cells were washed (day 0), and the untreated and treated cells were transferred upon reaching 85–95% confluence to 75 cm^2^ flasks and passaged at a 1:5 ratio. Untreated cells were transferred on day 0 to 75 cm^2^ flasks with a passage every two days until day 8. Cells treated with IdU were transferred on day 0 to 75 cm^2^ flasks with a passage on day 5 and cultured until day 9. The flasks treated with AzaC were transferred to 75 cm^2^ on day 2 and passaged every 2 days until termination on day 8. The cells treated with IdU + AzaC were transferred to 75 cm^2^ on day 4 and cultured until day 8.

All of the medium was replaced daily with fresh medium, and filtered supernatant (0.45 μm filter; Corning, New York, NY, USA, catalogue no. 430134) was collected and stored at −80 °C. Retrovirus induction was evaluated based on production of cell-free reverse transcriptase (RT) activity using the PERT assay.

### 2.4. PERT Analysis

Ten microliters of filtered supernatant were tested for RT activity using the PERT assay. Each sample was tested in triplicate at a 1:10 dilution (per previously described assay protocol [41]). Both the RT and PCR steps were performed in a Quantstudio 3 Real-Time PCR System (Applied Biosystems; Wilmington, DE, USA). The results are reported as pU/µL of the undiluted sample, calculated from a standard curve generated by testing serial dilutions of the HIV RT enzyme (Worthington Biochemical Corporation, Lakewood, NJ, USA) that was included in each assay. The linear regression of the standard curve had an R^2^ value > 0.99, and the values of the samples tested were within the linear range [39,41,42].

To test for retrovirus particles, two types of pelleted material were prepared from filtered supernatant and analyzed using the PERT assay. For one sample type, 1.3 mL of supernatant from untreated CHO-K1 cells and from the dual-treated day 8 sample (AzaC + IdU, 2.5 + 30 µg/mL) was centrifuged in an Optima Max-XP Ultracentrifuge (Beckman-Coulter, Sykesville, MD, USA; TLA-45 rotor) at 45,000 rpm for 90 min at 4 °C, and the pellet was resuspended in 25 µL F12K complete medium. Ten microliters of the resuspended pellet was diluted 1:10, 1:100, and 1:1000 in NZ + DTT Buffer [41]. The second sample type was processed similarly to the first, except the pellet was resuspended in the original volume of 1.3 mL and tested in the PERT assay at 1:10 and 1:100 dilution. Controls analyzed under the same conditions included complete medium and simian foamy virus type 2 stock grown in our laboratory in *Mus dunni* cells (SFV-2/MD, 10^5.03^ TCID_50_ per mL) using 1.8 µL (193 TCID_50_) and 5 µL (538.76 TCID_50_) in 1.3 mL of F12K media.

### 2.5. TEM Analysis

Cell pellets were prepared from cells treated with drug added at 16 h post-seeding. Cell pellets of 3–5 × 10^6^ cells were collected on day 7 for 2.5 µg/mL AzaC and on day 8 for 20 µg/mL IdU and for combined 2.5 µg/mL AzaC + 20 µg/mL IdU. Untreated cells were from the CHO-K1 master cell bank. Cells were fixed and analyzed by TEM using previously described protocols [32,43], except that the samples were examined with the JEOL JEM 1400 transmission electron microscope (JEOL Ltd.; Akishima, Tokyo, Japan). Digital images were taken with a Gatan 1000XP camera system (Gatan Inc.; Pleasanton, CA, USA).

### 2.6. Infectivity Analysis

For each cell line, five 25 cm^2^ flasks were seeded with the number of cells that would reach ~50% confluence at 24 h post-seeding. One millilitre of inoculum was added in a total volume of 5 mL of complete medium containing 4 μg/mL of polybrene (Millipore Sigma, St. Louis, MO, USA, catalogue no. TR1003G). The inocula were filtered supernatants collected from drug-treated cells (2.5 μg/mL AzaC, 30 μg/mL IdU, and 2.5 + 30 μg/mL for AzaC + IdU), complete medium containing polybrene as a negative control, and SFV-2/MD (193 TCID_50_, stock, TCID_50_ = 10^5.03^/mL) as a positive control. Vero cells were seeded at 5 × 10^5^ total cells and inoculated at passage 132; 293 cells were seeded at 1.33 × 10^6^ total cells and inoculated at passage 50; A549 cells were seeded at 5 × 10^5^ total cells and inoculated at passage 95; *M. dunni* cells were seeded at 2.5 × 10^5^ total cells and inoculated at passage 43; and MRC-5 cells were seeded at 5 × 10^5^ total cells and inoculated at passage 26. After 24 h incubation with the inoculum (day 0), cells were trypsinized and transferred to 75 cm^2^ flasks. Filtered supernatant (500 μL) was collected at each cell passage over the 25-day culture period (10 passages) and stored at −80 °C for PERT analysis. The positive controls were cultured until extensive cytopathic effect (CPE) was seen. At the end of the infectivity studies, cell pellets were prepared from each flask by washing trypsinized cells with Dulbecco’s phosphate-buffered saline (DPBS; pH 7.4, no calcium, no magnesium; Quality Biologics, Gaithersburg, MD, USA; catalogue no. 114057101) and storing in 200 μL containing 2.5 × 10^6^ cells at −80 °C for HTS analysis.

### 2.7. HTS Analysis

For virus integration analysis, short-read sequencing was performed on selected cell pellets from the infectivity studies. Total RNA was extracted from cells per protocol using a Qiagen RNeasy kit (Qiagen; Hilden, Germany; catalogue no. 69504). RNA was quantified using the Qubit Broad Range assay kit (Invitrogen, catalogue no. Q10210). Library construction and sequencing was performed by the CBER core facility. The libraries were prepared using the Illumina DNA preparation kit and sequenced using NovaSeq S1 flow cells (Illumina, San Diego, CA, USA, catalogue no. 20060060 and 20028317, respectively) for 2 × 151 cycles for the 293 cell samples and 2 × 101 cycles for the A549 cell samples. Samples of each cell line (inoculated with supernatant from CHO cells treated with AzaC + IdU (2.5 + 30 µg/mL) and control cells) were multiplexed. The adapter-trimmed reads were processed using CLC v22.0.2 and v23.0.4 (CLC Bio, Aahus, Denmark) with default parameters, except the quality limit was increased to 0.001 (equivalent to a Q-score of 30) and the minimum read length was 50. Initially, host sequences were removed using the CHO genome (GCF_000223135.1), and targeted bioinformatic analysis was performed by mapping the reads to 10 known CHO retrovirus genomes (NCBI GenBank: type C retroviral sequence accession numbers U09104, M9961, MN527961, MN527962, and type A retroviral sequence accession numbers X07947, M73970, M34949, M34950, M34951, MN527960) using parameters of sequence identity (sequence fraction) 0.9 and mapped read length (length fraction) 0.8. The identity of the hits was confirmed and identified as viral or cellular. For the infectivity study with the 293 cells, the negative control had a total of 1,688,152,336 (raw 2,429,162,810) reads, and the read length was 151 bp, and for the 293 cells inoculated with supernatant from AzaC + IdU-treated cells, the total number of quality reads was 979,644,910 and the read length was 151 bp. For the A549 untreated control, 1,360,260,880 quality reads were obtained with a read length of 101 bp; for the A549 inoculated with the supernatant from the AzaC + IdU treated cells, the total number of quality reads was 1,361,216,280, and the read length was 101 bp.

For characterization of the viruses, long-read sequencing was performed using the Oxford Nanopore MinION (Oxford Nanopore Technologies, Oxford, UK) on filtered supernatant that was collected on day 7 post induction of CHO cells with AzaC + IdU (2.5 + 30 µg/mL). Supernatant (2 × 10.4 mL) was pelleted by ultracentrifugation (Ultracentrifuge XE90, Beckman-Coulter; Sykesville, MD, USA using an SW 41 ti rotor) at 125,000× *g* for 4 h at 4 °C with the brake off. The pellets were resuspended in 500 µL of sample supernatant, and 250 uL was placed into two 1.5 mL tubes, resulting in 4 × 250 µL for each supernatant, and RNA was extracted using TRIzol (Invitrogen, Waltham, MA, USA, catalogue no. 10296010) per the manufacturer’s protocol, except 4 μL of linear acrylamide (Invitrogen, catalogue no. AM9520, stock 5 mg/mL) was added to help visualize the pellet, and samples were incubated at −80 °C overnight. The four pellets were dissolved in nuclease-free water (Quality Biologic, catalogue no. 351-029-721), pooled, and the total volume increased to 116 µL for DNase treatment to remove residual cellular DNA using 10 µL DNase (1000 u) and 14 µL of 10× DNase Buffer (Promega, Madison, WI, USA, catalogue no. M6101). Samples were incubated at 37 °C for 1 h. DNase was removed using a Zymogen RNA clean-up kit (Zymo Research, Orange, CA, USA, catalogue no. r1018) according to protocol. DNA and RNA were quantified by the Qubit 3 (Invitrogen) using their high-sensitivity DNA (Invitrogen, catalogue no. Q33230) and RNA (Invitrogen, catalogue no. QS32852) kits. Double-stranded cDNA was generated using the Maxima H Minus Double-Stranded cDNA Synthesis Kit (Thermo Fisher Scientific; Waltham, MA, USA, catalogue no. K2562, K2563) using random hexamers according to the manufacturer’s protocol. The cDNA was then purified using the GeneJET PCR Purification Kit (Thermo Fisher Scientific, catalogue no. K0701, K0702) according to protocol and then used as input for library preparation and sequencing using the ligation sequencing kit v14 (Oxford Nanopore Technologies, Oxford, UK, catalogue no. SQK-LSK114) following the manufacturer’s instructions. Sequencing was performed on R10.4.1 flow cells (Oxford Nanopore Technologies, catalogue no. FLO-MIN114) on a MinION Mk1B for 30 h.

Sequences were base called with DORADO version 0.9.0 (Oxford Nanopore Technologies) using the duplex mode (dna_r10.4.1_e8.2_5khz_stereo@v1.3) and the super accurate algorithm (dna_r10.4.1_e8.2_400bps_sup@v5.0.0) with quality trimming set at Q7 (dorado duplex -v sup -min-qscore 7). The duplex reads were processed with samtools view to remove simplex reads that have duplex offspring such that only simplex (dx:i:0) and duplex (dx:i:1) reads remained (1,102,625 reads). Reads less than 50 base pairs were removed using Cutadapt [44], resulting in 1,102,241 reads, with an N50 read length of 3562, a mean read length of 1965, and a mean read quality of 18.92.

The 1,102,241 reads were aligned using blastn (E-value 1e-5, max_hsps 10) against a collection of sequences indicated in Table 1 to remove nonviral sequences.

1,025,038 reads were removed, resulting in 77,203 reads remaining for subsequent analysis. The 77,203 reads were analyzed using blastn (E-value 1e-4; max_hsps 10) against U-RVDBv29, modified to contain only a single SARS-CoV-2 reference sequence to facilitate the efficiency of the analysis. This resulted in 14,027 reads that had hits to 14,305 reference viral genomes in RVDB. The 14,027 reads were aligned against the 14,305 references using minimap2 (using default parameters minimap2 -x map-ont -a -t 40). This resulted in 168 hits based on the default alignment parameters with Minimap2. Analysis of these hits, i.e., excluding low complexity and non-viral hits, resulted in the 10 hits listed in Table 3 below (Section 3.5). Furthermore, the same results were obtained when the 77,203 reads were aligned with the 14,305 references using Minimap2. Additionally, the 77,203 reads were analyzed by blastn and tblastx against the NCBI nt database (downloaded in November 2024) to look for potentially unknown or distantly related endogenous sequences.

## 3. Results

### 3.1. Determination of Cell Growth Characteristics and Optimization of Virus Induction Conditions

**Figure 1:**
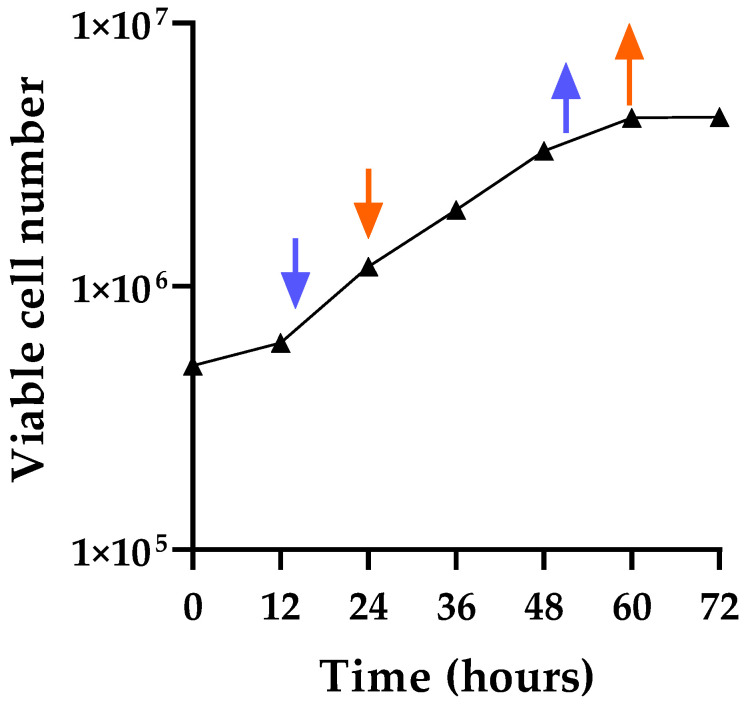
CHO-K1 growth curve and drug testing times. CHO-K1 cells were seeded at time 0 with 5 × 10^5^ cells, and the total number of viable cells was counted at 12 h, 24 h, 36 h, 48 h, 60 h, and 72 h post-plating to generate a growth curve. Two flasks were set up for each time point, and four independent viable cell counts were obtained for each flask. The average counts are shown for each time point. The arrows indicate the times tested for optimizing the induction conditions: blue indicates adding the drug at 16 h and removing it at 52 h, and red indicates adding the drug at 24 h and removing it at 60 h.

The growth curve was obtained for the CHO-K1 cells to determine the optimal times for adding and removing the drug. The PDT was calculated from the linear log phase (12 h to 60 h) as 21 h, which was similar to the 24 h that has been generally described for CHO cells. Our previous studies for optimizing induction conditions indicated that the drug should be added in the early log phase, and cells should be treated for at least one PDT before the drug is removed [45,46]. The growth curve for CHO-K1 cells is shown in Figure 1, which indicates the early log phase was around 16 h to 24 h for adding drug.

**Figure 2:**
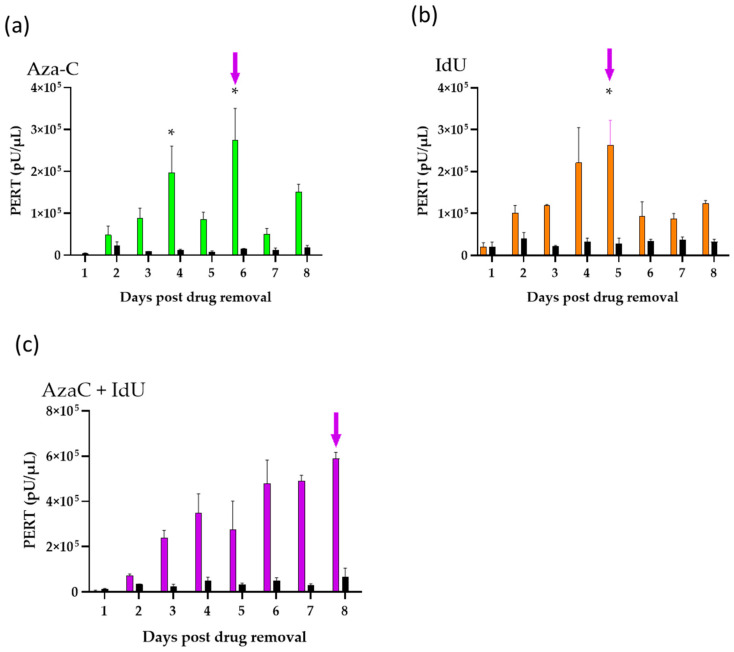
Analysis of virus induction by drug treatment in CHO-K1 cells. Cells were treated with drugs for virus induction, and the PERT assay was used for measuring the RT activity in cell-free supernatant. (**a**) AzaC (2.5 µg/mL). (**b**) IdU (30 µg/mL). (**c**) 2.5 µg/mL AzaC + 30 µg/mL IdU. Untreated CHO-K1 cells were included as a control (black) to determine the background level of RT activity. Arrows indicate the day of supernatant used for the infectivity experiments (below), and an asterisk indicates the day of cell passage.

To determine the optimum conditions for endogenous retrovirus induction from CHO-K1 cells, drug (2.5, 5.0, 7.5, or 10 µg/mL for AzaC and 10, 30, or 50 µg/mL for IdU) was added at 16 h and 24 h (corresponding to the beginning and early log phase, respectively) and removed at 52 h and 60 h, respectively, to treat the cells for 1.7 PDT. The times for adding and removing the drugs are indicated in Figure 1. Cells were cultured for up to 7 days, and virus induction was evaluated daily based on the RT activity in filtered supernatant using the PERT assay. The optimized drug treatment conditions were used for the virus induction studies described below (Figure 2).

### 3.2. Evaluation of Endogenous Retrovirus Induction from CHO-K1 Cells

Previous studies have shown induction of endogenous retroviruses by chemical treatment of rodent cell lines [10,19,42,46,47,48,49,50,51]. Furthermore, optimization of endogenous retrovirus induction conditions using AzaC and IdU was shown for rodent and nonrodent cell lines [42,45,46]. We applied the optimized virus induction strategy and previously tested drug concentrations to evaluate endogenous virus activation from the CHO-K1 cells. In this study, the optimized conditions for endogenous retrovirus induction for single-drug treatment were determined by testing a range of drug concentrations added at two time points based on the growth curve (as described in the previous section). Since earlier studies indicated dual drug treatment could enhance endogenous virus production [42,45], single and dual drug treatments were evaluated for endogenous retrovirus induction from CHO-K1 cells. Optimized conditions were determined based on drug cell toxicity, cell recovery, and PERT activity induced from the cells. Based on the results, drugs (2.5 µg/mL AzaC, 30 µg/mL IdU, and AzaC [2.5 µg/mL] + IdU [30 µg/mL]) were added at 16 h post seeding the cells and removed after 1.7 PDT (at 36 h) as described in Section 2. The entire medium was replaced daily and analyzed using the PERT assay to accurately detect the level of virus induced from the cells. Virus induction was measured by testing filtered supernatant that was collected daily and pooled from four flasks in order to generate sufficient inoculum for the infectivity studies described below. The results are shown in Figure 2. As expected, a decrease in RT activity was seen following cell passage.

The results shown in Figure 2 are representative of the trend in PERT activity seen with drug treatment in three other independent induction studies. This included an experiment where the cells were treated with AzaC 2.5 µg/mL + IdU 20 µg/mL combined or with these drugs separately using a lower concentration of IdU to reduce cell toxicity.

In general, a greater log of RT activity was seen due to virus induction in the drug-treated versus untreated cells. Overall, greater toxicity was seen with IdU and dual-treated IdU + AzaC cells (60–85% confluence on day 8) compared with the untreated and AzaC-treated cells, based on morphology and cell growth. Using IdU at 20 µg/mL, the cell toxicity was slightly lower, and a similar profile was seen for the RT activity.

To determine that the RT activity was associated with virus particles, filtered supernatant collected on day 8 from dual-treated (AzaC 2.5 µg/mL + IdU 30 µg/mL) cells and supernatant from day 7 untreated cells were concentrated by ultracentrifugation 52-fold, and serial dilutions (10, 100, and 1000 times) were analyzed using the PERT assay (Table 2). Both the untreated and dual-treated resuspended, pelleted material showed assay interference, most likely due to the presence of concentrated proteins in the medium, since the RT activity was greater in the 100× dilution compared to the 10× dilution. The dilution-adjusted RT activity in the original supernatant of the untreated sample was around 1.9 × 10^6^ pU/µL, and the dual treated was around 5.7 × 10^8^ pU/µL.

**Figure 3:**
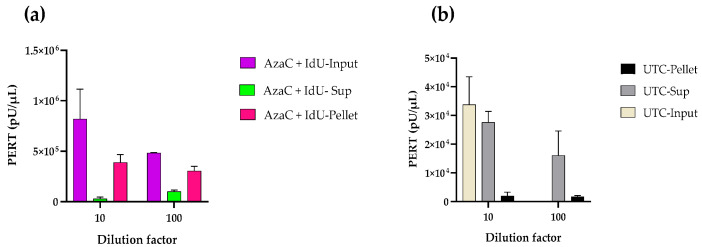
Evaluation of particle-associated RT produced from CHO-K1 cells. A second pelleting experiment was carried out, but instead of resuspending the pellet in 25 µL of media to concentrate the pellet, it was resuspended in 1.3 mL, the input volume. PERT analysis was performed on (**a**) resuspended pellet and supernatant obtained from cells that were dual treated with 2.5 µg/mL AzaC + 30 µg/mL IdU and (**b**) supernatant from untreated cells (UTC). The samples were tested at 1:10, as per normal PERT protocol, or 1:100 in NZ + DTT buffer. The PERT activity (pU/µL) indicated for the original undiluted material.

In a second experiment, pellets from day 8 of the dual-treated cell supernatant and day 7 of the untreated cell supernatant were resuspended after centrifugation in the same volume as the input material (1.3 mL). The supernatant from the ultracentrifugation was retained to determine the soluble RT activity remaining in the supernatant after pelleting the particles. The results from the PERT analysis of these samples diluted 10× and 100× are shown in Figure 3. The PERT activity in the starting dual treated material was around 8 × 10^5^ pU/µL. Higher RT activity was detected in the resuspended pellet compared to that remaining in the supernatant after ultracentrifugation. The PERT activity in the starting supernatant from untreated cells was around 3 × 10^4^ pU/µL; however, most of the RT was soluble, and little was in the pelleted material. The results further confirm the induction of RVLPs in the drug-treated cells. In an additional experiment under the same centrifugation conditions, the PERT results for the culture medium were negative and were positive for SFV2/MD.

### 3.3. TEM Determination of Virus Particles

**Figure 4:**
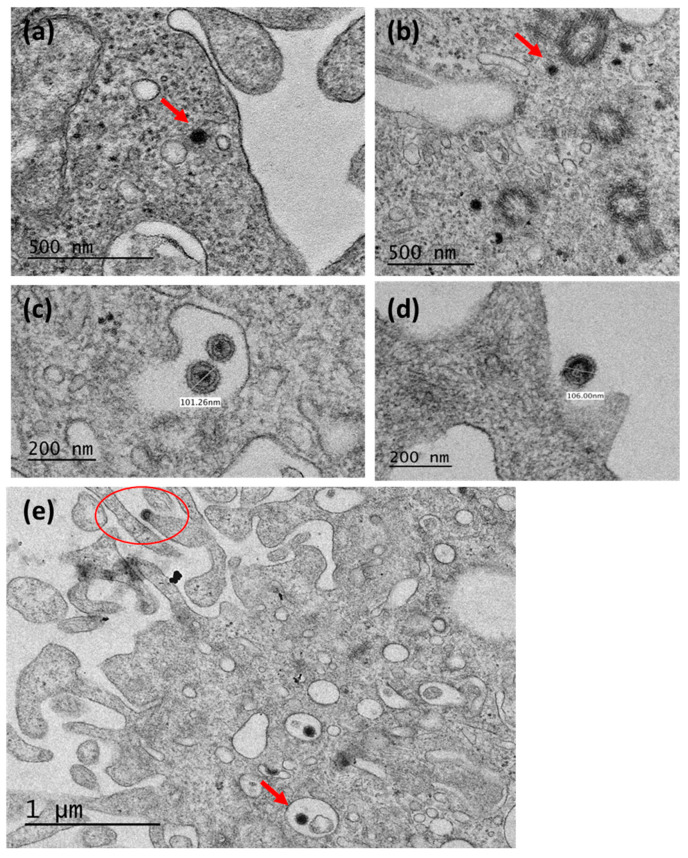
TEM analysis of retroviral particles in CHO-K1 cells. (**a**) Untreated cells from the CHO-K1 master cell bank. Cells treated with AzaC (2.5 µg/mL) and collected on day 7 show (**b**) intracytoplasmic type A particles and (**c**) mature type A/B virus particles. (**d**) Type C particles in cells treated with the IdU (20 µg/mL) and collected on day 8. (**e**) Type A and type C particles in cells treated with AzaC + IdU (2.5 µg/mL AzaC + 20 µg/mL IdU) and collected on day 8. The arrow in (**a**,**b**,**e**) indicates type A particles, and the circle in (**e**) indicates a type C particle. Scale is shown in each panel, and particle size is indicated in panels (**c**,**d**).

Cell pellets were analyzed by TEM from untreated and drug treated cells to evaluate for the presence of retroviral particles. As expected from the PERT activity, an increase in viral particles was seen in the drug treated cells compared with the untreated cells (Figure 4).

AzaC has previously been shown to induce primarily type A particles [6,8,9,10,19,21,52]. In our studies, induction of intracytoplasmic type A particles (about 70–80 nm in diameter) was seen based on the higher number of particles compared to untreated cells (Figure 4b compared to Figure 4a). As shown in a previous study, these were seen near microtubules and centrioles, exhibiting an electron-dense core. Additionally, intracisternal type A particles were seen budding in the endoplasmic reticulum with a diameter of about 100 nm (Figure 4c).

IdU has been shown to induce type C particles [49,51]. Figure 4d shows IdU treatment of CHO-K1 cells induced budding type C particles (106 nm diameter) with an envelope and icosahedral/ovoid-like capsid. No type C particles were observed in the untreated cells even upon extensive investigations. The increase in the type A particles compared to the type C particles seen in the drug-treated cells is reflected by the lower number of potentially infectious type C genomes present in the CHO cells and also the lower copy number of type C versus type A sequences in the CHO cell genome: 100–300 copies per genome of type C [11,17] compared to 10^2^–10^3^ copies of type A per genome [24,25]. Dual drug treatment of cells induced both type A particles that were seen in the cytoplasm (Figure 4e) and some near microtubule assembly centres, and budding type C particles at the plasma membrane. The results further indicated that the RT activity produced from the CHO-K1 cells was mainly due to type A particles and that fewer particles seen in the untreated cells were reflected by the lower RT activity compared to drug-treated cells (~10^4^ in control samples versus 10^5^ in drug treated samples).

### 3.4. Infectivity Analysis of CHO RVLPs

**Figure 5:**
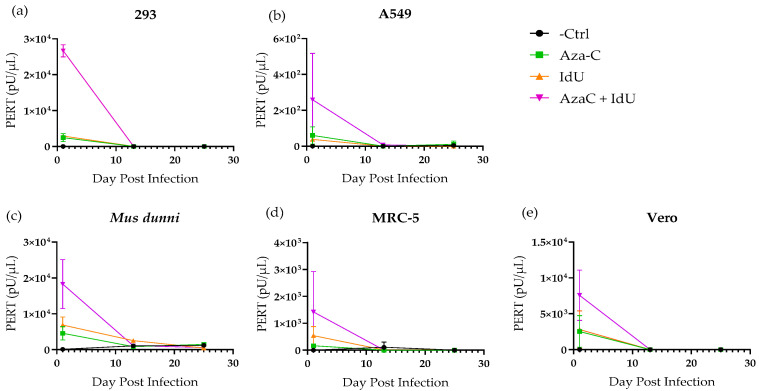
Infectivity analysis of CHO RVLPs. Cell lines (293, A549, *M. dunni*, MRC-5, and Vero; panels (**a**–**e**), respectively) were inoculated with filtered supernatant from drug-treated and untreated CHO-K1 cells. Samples were tested by the PERT assay at each cell passage, and the RT activity was reported as pU/uL, present in the original sample. Results (with standard deviation of triplicate assay samples) are shown on selected samples tested to evaluate virus replication during the cell culture period. The last day for each cell line indicates the termination of the experiment.

To evaluate if the induced RT activity was associated with infectious retroviruses, filtered supernatant from drug treated CHO-K1 cells (AzaC day 6, IdU day 5, and dual treated day 8) was used to inoculate cell lines of different species, which included human 293, A549, and MRC-5 cell lines; African green monkey Vero; and wild mouse *M. dunni* cells. These were used to provide a broad host range of target cell species, since the envelope region in CHO type C RVLPs had homology with amphotropic MLVs, which can infect mouse and nonrodent cell lines, including human. Cells were also mock-infected with medium as a negative control. Samples were collected at each cell passage to evaluate virus replication based on the increase in PERT activity during the extended culture time. Initial testing was performed to compare the increase in RT activity at mid and terminal points with input RT activity present in the inoculum at 24 h post infection, when the medium was removed and cell culture continued. Test samples, including the negative and positive controls, were analyzed in the same PERT assay for each cell line. In all cases, high RT activity was only seen due to the input inoculum (Figure 5). As expected, based on the induction experiments (Figure 2), the RT activity was higher in the inoculum from the dual treated cells (around 10^3^–10^4^ pU/µL) compared to the IdU- or AzaC-treated cells. This was also true of the A549 cells, but the inoculum RT activity was lower by 10–100-fold than that for the other cell lines. The results were confirmed in a repeat of the PERT assays. In all cases the RT activity did not increase during cell propagation but decreased to levels similar to the negative control over the extended culture time until termination at day 25. The results with the drug treated inocula indicated a lack of virus replication associated with endogenous, inducible RVLPs in CHO cells.

SFV-2 was selected as a positive control for all of the cell lines since it is known to broadly infect cells of a variety of host species [53]. The results are shown in Figure S1. Additionally, progressive CPE and cell death were seen with increasing RT activity, which is generally expected with SFV-2 infection, with differences in kinetics of virus replication depending on the cell type and species of cell origin [53,54]. SFV-2 infection of Vero cells resulted in a low level of RT activity, above the negative control, with similar kinetics as previously obtained [54].

Our previous induction studies with single or dual treatment of mouse cells produced an early peak of RT activity at days 2–3, which decreased thereafter [42,45]. In this study, dual treatment of CHO-K1 cells resulted in increased RT activity that was sustained throughout the culture period (Figure 2c). To investigate the potential induction of a replicating ecotropic retrovirus, filtered supernatant from day 8 of the dual treated cells was inoculated onto CHO-K1 cells, and supernatant was collected for PERT analysis at each cell passage up to day 29 (12 passages). At the last cell passage, four flasks were set up for the cells inoculated with the drug treated sample and three set up with the negative control. The cell numbers and PERT activity were determined for each flask to calculate the RT activity per cell for comparing any increase in RT activity due to a replicating virus with the RT activity produced from the negative controls. The results showed no significant increase in RT activity compared to the control.

The PERT assay showed no evidence that a retrovirus induced from the CHO-K1 cells was able to replicate in the target cells used in the infectivity studies. To further investigate if any retroviral sequences were able to enter and integrate into the target cell genome, HTS analyses were performed on pellets prepared at the termination of the culture period (day 25) from 293 and A549 cells inoculated with supernatant from dual treated CHO cells and from untreated control CHO cells. Analysis of short-read HTS data using CLC genomics workbench with stringent mapping criteria found no reads that mapped to any of the 10 reference virus genomes, which were identified to be induced in the dual treated CHO cells (described in the next section and indicated in Table 2). Overall, the results of the infectivity studies using selected target cells indicated the absence of infectious, inducible retroviruses from the CHO-K1 cell line.

### 3.5. Characterization of Inducible CHO RVLPs Using Long-Read HTS

Long-read sequencing was performed using the Oxford Nanopore Technology to identify the type of retroviral particles that were associated with the high RT activity present in day 7 supernatant from dual drug-treated cells (Figure 2c). Ten viral reference genomes were identified by blastn analysis using RVDB, which included CHO type A and C viruses (shown in Table 3). Furthermore, blastn against NCBI nt did not find any additional viral sequences, and tblastx did not find any distantly related viral sequences.

The consensus full genome sequences of the 10 virus sequences generated from the long-read sequencing had 90–99% identity to the published reference genomes.

**Table 3:** Identification of RVLPs induced in dual drug-treated CHO-K1 cells using long-read HTS analysis.

Ref. Seq. ID *	Description	Ref. Seq Length (bp)	Minimap Alignment to the 77K Reads vs. Each Ref Independently	
			Read No.	% Genome Length Covered
M34949	C. griseus intracisternal A-particle retrovirus like sequences.	1953	139	81.7
M34950	C. griseus intracisternal A-particle retrovirus like sequences.	1570	112	86.6
X07947	Chinese hamster Intracisternal A-Particle gene (IAP) corresponding to 5′LTR-GAG	2061	58	99.2
M73970	Chinese hamster provirus gene, complete cds (IAP)	6404	261	83.0
M34951	C. griseus intracisternal A-particle retrovirus like sequences. (IAP)	2186	103	99.8
MN527960	Cricetulus griseus endogenous virus CHERV-1b cell line CHO-K1, complete sequence (IAP)	7924	402	100
MN527961	Cricetulus griseus endogenous virus CHERV-2g cell line CHO-K1, complete sequence. (Type C)	8704	2742	100
M99691	Hamster retroviral sequence mRNA (Type C)	1184	1292	100
U09104	Cricetulus griseus 5′ long terminal repeat, complete sequence; gag, pol, and env genes, complete sequence; and 3′ long terminal repeat complete sequence. (Type C)	9603	1151	81.9
MN527962	Cricetulus griseus endogenous virus CHERV-3g cell line CHO-K1, complete sequence (Type C)	8340	874	100

* Genbank accession number.

## 4. Discussion

Human health and safety are paramount when releasing biologically derived products for public use. Therefore, extensive and redundant testing is recommended using various biological, biochemical, and molecular assays at different stages during the manufacturing process to assure the absence of adventitious agents in the final product [55]. Although CHO-K1 cells are known to produce retroviral particles, this cell line and its derivatives are a popular cell–substrate for the production of biotherapeutic protein products due to the restricted susceptibility of the cell line to infection by human viruses [56] and easy scale-up to produce proteins with human-like glycosylation and other post-translational modifications [3]. In the case of highly purified products, the potential concerns regarding CHO cells producing endogenous retroviruses can be addressed by incorporation of viral clearance steps and use of model viruses to demonstrate removal of known and unknown viruses by the manufacturing process [57]. However, there is increased interest in using CHO cells for the development of vaccines and gene therapies, where in some cases it may not be possible to incorporate rigorous virus clearance steps during manufacturing in order to retain product potency. Although there is a long-standing safety record using CHO-cell based products, potential safety concerns remain due to the previous, albeit rare, occurrences of virus contamination [58]. Furthermore, there is a lack of comprehensive characterization of endogenous retroviral sequences in the CHO cell genome. Moreover, as advances in high-throughput sequencing technology have been progressing, the scientific community has been discovering more and more dormant retroviruses and other endogenous viruses in the genomes of various species. It is known that CHO cells produce RVLPs that have been described morphologically as type A particles [6,7,8,10,11,12,25] and type C particles [12,14,16,17,47,48,49,59]. Since the CHO type C particles are related to MLV sequences, which can generate novel, recombinant retroviruses [16,17,38,60,61,62], it is important to investigate the host range of CHO RVLPs due to their relatedness in the env region to amphotropic MLVs.

Given the increase in the use of CHO cells for the production of various biologics [2,3,4] along with the advances in scientific knowledge, techniques, and technology over the last 30 years, we sought to extensively characterize RVLPs produced from CHO-K1 cells. To assess the potential for activation of an infectious, endogenous retrovirus due to stress on the cells during manufacturing, we used IdU and AzaC drug treatment to induce latent retroviruses and tested for infection and replication in various mammalian cell lines, including mouse (*M. dunni*), monkey (Vero), and human (293, A549, and MRC-5) that have broad susceptibility to retroviruses and other viruses. Using the highly sensitive PERT assay, induction of virus particles with AzaC and IdU was seen well above the basal level of RVLPs produced by the CHO-K1 cell line. Induction of retroviral particles from CHO-K1 cells was further demonstrated by pelleting the RT activity from cell-free supernatant, identifying CHO retroviral sequences by long-read HTS, and identifying RVLPs by TEM. Interestingly, all of the currently known CHO type A and type C retroviral sequences (Table 3) were identified by long-read sequencing, and no novel viral sequences were detected. Furthermore, in our study, it was noted that a larger number of reads mapped to type C virus genomes compared to type A, indicating the greater production of type C RVLPs. Duroy and colleagues [17] analyzed the CHO-K1 genome assemblies from published databases along with their own sequences. Based on a phylogenetic analysis using the *pol* region, they reported three groups of type C virus sequences. Group 1 consists of endogenous retroviral sequences that could potentially encode functional viruses based on expressing full-length transcripts with open reading frames for *gag*, *pol*, and *env* proteins. This group included a functional endogenous retrovirus termed ETC109F. Groups 1 and 2 formed a sister group but group 2 sequences had two stop codon mutations and three frameshift mutations and therefore were unlikely to form functional particles. Group 3 type C retroviral sequences were distinct, with two large deletions present in the *pol* region (totaling over 2300 bp) that might be transcribed but not produce a functional virus. In their analysis, U09104 was a group 3 type C virus. Our analysis indicated that MN527961 was identical to ETC109F, with 2742 reads mapping to the MN527961 reference virus genome (Table 3). It is noted that a high number of reads mapped to the type C reference genomes, supporting that the type C endogenous retroviral loci are actively expressed as viral particles.

Using cell-free supernatant from CHO-K1 cells treated with single or combined drugs, there was no evidence of replication based on searching for virus production using the highly sensitive PERT assay over an extended culture period or integration of RVLPs by HTS analysis of inoculated 293 and A549 cells. These results are similar to those previously reported. Emanoil-Ravier and colleagues used induction reagents on CHO-K1 and tested the infectivity of induced RVLPs on a panel of canine, rodent, simian, and human target cells and saw no signs of infection [19]. Dinowitz and colleagues purified RVLPs using ultracentrifugation and sucrose gradients and tested the RVLPs infectivity on a panel of human (A549, RD), rhesus (FRhL-1), bat (TB1Lu), mink (Mi 1LU), dog (Cf2Th), Chinese hamster (Dede, Don), and Syrian hamster (HaK, BHK-21) cells and found no signs of infection [18]. Hojman and colleagues induced CHO-HB cells with IdU and AzaC, as well as other inducers, to induce RVLPs and tested their infectivity on a large number of target cells from humans, rodents, monkeys, and bats and found no signs of infection and even saw no increases in oncogene markers that would indicate increases in A-particle translocation activity [51]. Shepherd and colleagues tested various cell banks, CHO cell banks among them, and found low levels of type C particles and A-particles produced (much lower than related cell banks), and no signs of infection were found in their target cells: *Mus dunni*, MRC-5, RD, and SC-1 cells [12]. Our results extend the previous studies by using sensitive, new assays such as the PERT assay and HTS analysis to demonstrate the absence of an infectious, latent retrovirus in the CHO-K1 cell line.

Rodent cell lines express endogenous retroviral particles, and CHO-K1 cells produce type A intracytoplasmic particles and type C particles that bud from the cell membrane and share a common ancestor with MLVs [17]. Type A particles are smaller RVLPs usually found in the cytoplasm of cells associated with mitotic and microtubule components, especially centrioles, that feature an electron dense centre and can vary in diameter depending on maturity (~70 nm for immature A-particles and 100–120 nm for mature A-particles) [7,8,10]. We could visualize these particles in our TEM pictures (Figure 4b,e) as well as the mature type A particles (usually larger with a recognizable membrane and spikes, electron dense nucleoid, and an intermediate shell; Figure 4c). We were also able to identify some A-particles that were pelleted from our supernatant, which matched full-length A-particles and/or degraded A-particles (Table 3). While these could act as endogenous mutagens by transposition to new locations in the genome at both the somatic cell and germline level [11,27,28,29,30,31,63], A-particles are highly unlikely to be infectious given the large number of mutations in their genome. However, type C RVLP sequences shared homology to MLVs, and as such, non-infectious and defective genomes can interact to generate infectious recombinant viruses. Of note is the homology of the RVLP env and retroviruses with a broad host range, such as amphotropic MLVs and feline leukemia retrovirus subgroup B (FeLV-B), which suggests RVLPs may have the potential for infecting non-rodent cells [16,17,33,34,35,36,38].

Endogenous retrovirus activation by chemical treatment of cells has been used for retrovirus discovery and characterization. There have been various earlier studies using drug treatment for inducing endogenous retroviral particles in CHO cells such as CHO-K1 [10,14,19,47,48] and CHO-HBs [51], and other rodent cell lines like N4 mouse neuroblastoma cells [50] and K-BALB [64,65,66]. Our laboratory developed an optimized strategy for chemical induction and using the sensitive PERT assay for retrovirus detection from K-BALB cells [42,67]. Furthermore, this strategy was applied for induction and infectivity studies of endogenous retroviruses in African green monkey Vero cells using similar approaches [45,68]. It is noted that the effectiveness and level of endogenous retrovirus activation were cell-line and perhaps species dependent: CHO cells were more sensitive to toxicity by IdU than K-BALB, whereas Vero cells were fairly resistant. In contrast, AzaC was more toxic to Vero cells than to rodent cells.

Considering the accumulated data for the lack of evidence of infectivity for CHO RVLPs, assessment for potential infectious retrovirus and risk management strategies should be maintained for product manufacturing using rodent cell lines. This is due to potential unexpected exposure of the facilities or reagents to rodents based on historical cases [58,69]. Since CHO-K1 cells are broadly used in research and biopharmaceutical applications, the potential for recombination involving endogenous retroviral sequences also needs to be considered [61,62,70].

## 5. Conclusions

The results of this study showed no evidence of infectious RVLPs constitutively produced or chemically induced from CHO-K1 cells. It is recognized that our results are based on infection of the selected target cells and are limited to in vitro studies. Therefore, due to the relatedness of the CHO-K1 RVLPs and MLV sequences, which are known to generate novel recombinant viruses, it is prudent to continue vigilance using the cells for biomanufacturing, to address the theoretical possibility of the generation of an infectious virus involving RVLP sequences.

## Figures and Tables

**Table 1:** Sequences for filtering nonviral sequences.

GenBank Accession No.	Description
none	DNA_CS, control lambda DNA (Nanopore)
DQ222453.1	Bos taurus 18S ribosomal RNA gene, internal transcribed spacer 1, 5.8S ribosomal RNA gene, internal transcribed spacer 2, and 28S ribosomal RNA gene, complete sequence
J00053.1	Chinese hamster Alu-equivalent repeat (clone 34) DNA
J00054.1	Chinese hamster Alu-equivalent repeat (clone 49a) DNA
J00055.1	Chinese hamster Alu-equivalent repeat (clone 49b) DNA
J00056.1	Chinese hamster Alu-equivalent type 2 repeat (clone 49c) DNA
J00057.1	Chinese hamster Alu-equivalent repeat (clone 49d) DNA
J00058.1	Chinese hamster Alu-equivalent repeat (clone 63) DNA
J00628.1	mouse b1 ubiquitous repeat (copy a) mRNA and flanks
J00631.1	mouse b1 ubiquitous repeat (copy b) mRNA and flanks
J00632.1	mouse b1 ubiquitous repeat (copy c) mRNA and flanks
KY962518.1	Homo sapiens external transcribed spacer 18S ribosomal RNA gene, internal transcribed spacer 1, 5.8S ribosomal RNA gene, internal transcribed spacer 2, 28S ribosomal RNA gene, external transcribed spacer, and CDC27-like protein pseudogene, complete sequence
NC_007936.1	Cricetulus griseus mitochondrion, complete genome
NM_001244575.1	Cricetulus griseus actin beta (Actb), mRNA
NR_046239.1	Rattus norvegicus 45S pre-ribosomal RNA (Rn45s), ribosomal RNA
NR_046263.1	Cricetulus griseus 45S ribosomal RNA (Rn45s), ribosomal RNA
X52123.1	Cricetus cricetus mRNA for glyceraldehyde-3-phosphate dehydrogenase (GAPDH) (EC 1.2.1.12)
XM_027388602.2	PREDICTED: Cricetulus griseus alpha tubulin acetyl-transferase 1 (Atat1), transcript variant X1, mRNA
XM_027427709.2	PREDICTED: Cricetulus griseus keratin 16 (Krt16), mRNA
XM_027427711.2	PREDICTED: Cricetulus griseus keratin 14 (Krt14), transcript variant X1, mRNA
XR_005974209.1	PREDICTED: Microtus oregoni 5S ribosomal RNA (LOC121447662), rRNA
U78038.1 (nt 1-1164 and nt 1222-2068)	Mus musculus beige gene, LINE 1 repetitive element
U09104.1 (nt 83-344) and nt 8768-8892)	CHO SINE element sequences
In-house	CHO_related 7SL sequence

**Table 2:** PERT activity in pelleted supernatant ^a^ from dual drug-treated and untreated samples.

Sample	Dilution Factor	Cq ^b^ Average	RT (pU/µL) Average
AzaC + IdU (2.5 + 30 µg/mL) treated cells: d8 resuspended pellet	10×	27.48	339,767.33
	100×	22.58	5,755,904.90
	1000×	26.95	325,327.98
Untreated cells: d7 resuspended pellet	10×	33.18	5735.42
	100×	31.22	19,729.09
	1000×	35.25	1482.53

^a^ An amount of 1.3 mL of sample was ultracentrifuged, and the pellet resuspended in 25 µL. ^b^ C_q_ or Ct denotes the quantification cycle or cycle threshold.

## Data Availability

Data can be provided upon request.

## Supplementary Materials

The following supporting information can be downloaded at: https://www.mdpi.com/article/10.3390/v17111408/s1, Figure S1: Positive control results for infectivity studies of CHO RVLPs.
