# Supplementary material for: Characterization of Chemically-Induced Endogenous Retroviral Particles in the CHO-K1 Cell Line
Source: Viruses. 2025 Oct 23;17(11):1408. doi: 10.3390/v17111408 (PMC12656867; doi:10.3390/v17111408)
Supplement: Supplementary file 1 [file viruses-17-01408-s001.zip › viruses-3752882-supplementary.pdf]

Supplementary materials

Characterization of chemically-induced endogenous retroviral particles in the CHO-K1 cell line

Nicholas B. Mattson [1], Trent J. Bosma [†], Yamei Gao [2], Sandra M. Fuentes [2], Pei-Ju Chin [2], and Arifa S. Khan [2,*]

1 University of Delaware, National Institute for Innovation in Manufacturing Biopharmaceuticals, 590 Avenue 1743, Room 661B, Newark, DE 19713, USA
2 Laboratory of Retroviruses, Division of Viral Products, Office of Vaccines Research and Review, Center for Biologics Evaluation and Review, U.S. Food and Drug Administration, Silver Spring, MD 20993, USA; Yamei.gao@fda.hhs.gov (Y.G.); Sandra.Fuentes@fda.hhs.gov (S.M.F.); Pei-ju.chin@fda.hhs.gov (P-J.C.)
† Current Address: Silver Spring, MD 20904, USA; tjbosma@gmail.com
* Correspondence: arifa.khan@fda.hhs.gov; Tel.: +1-240-402-9631

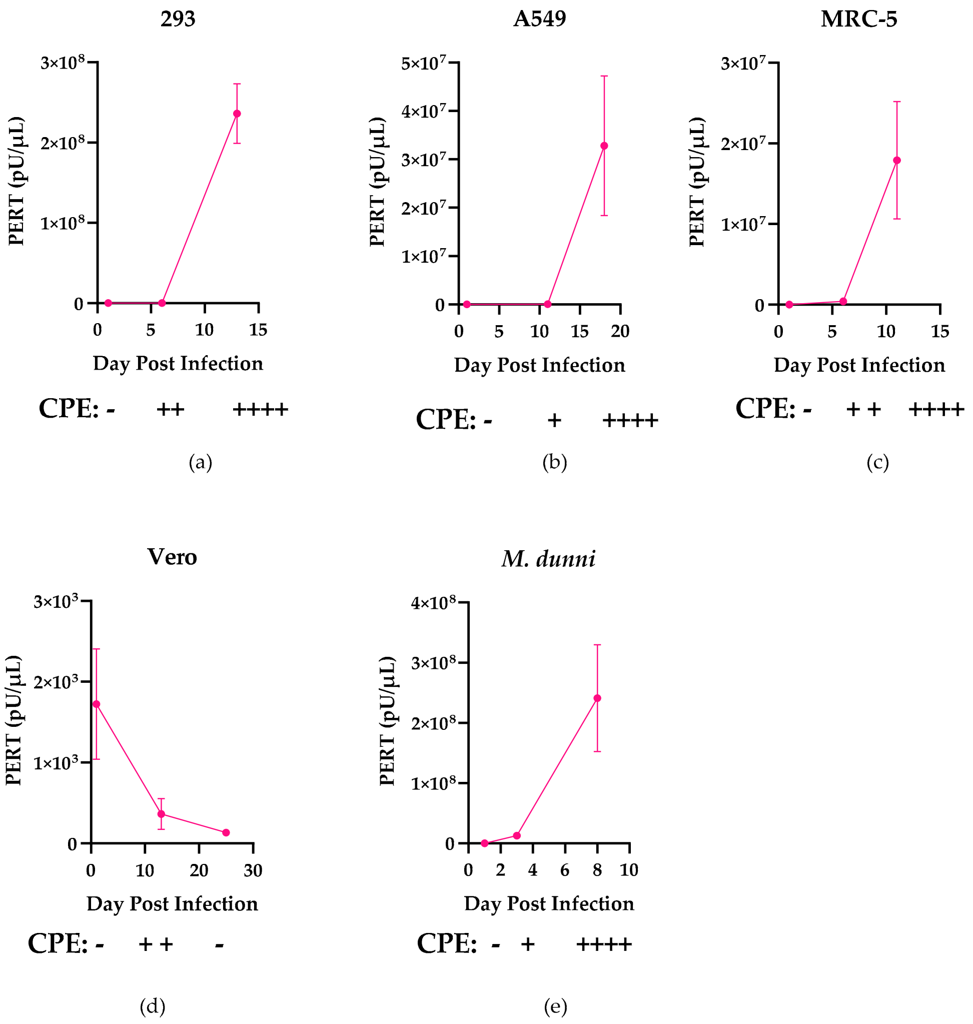

Figure S1. Positive control results for infectivity studies of CHO RVLPs. SFV-2 (193 $TCID_{50}$/mL) was inoculated as positive control for 293, A549, MRC-5, Vero, and *M. dunni* (a – e, respectively). The results of the PERT activity are shown with standard deviation for triplicate samples for testing filtered supernatant at various times during the cell culturing (early, mid, and culture termination). CPE was noted by microscopic observation of the cell monolayer: -, no CPE; +, less than 25% CPE; ++, 25-50% CPE; +++, 50-75% CPE; and ++++, more than 75% CPE. The positive controls were set up in the same experiment as those shown in Figure 5 and therefore the results of the negative control cells were the same.
